# Targeting the Nerve Growth Factor Signaling Impairs the Proliferative and Migratory Phenotype of Triple-Negative Breast Cancer Cells

**DOI:** 10.3389/fcell.2021.676568

**Published:** 2021-06-29

**Authors:** Marzia Di Donato, Giovanni Galasso, Pia Giovannelli, Antonio A. Sinisi, Antimo Migliaccio, Gabriella Castoria

**Affiliations:** ^1^Dipartimento di Medicina di Precisione, Università degli Studi della Campania “Luigi Vanvitelli”, Naples, Italy; ^2^Dipartimento di Scienze Mediche e Chirurgiche Avanzate, Università degli Studi della Campania “Luigi Vanvitelli”, Naples, Italy

**Keywords:** triple-negative breast cancer, NGF, TrkA, NGF signaling, new therapy

## Abstract

Triple-negative breast cancer is a heterogeneous disease that still lacks specific therapeutic approaches. The identification of new biomarkers, predictive of the disease’s aggressiveness and pharmacological response, is a challenge for a more tailored approach in the clinical management of patients. Nerve growth factor, initially identified as a key factor for neuronal survival and differentiation, turned out to be a multifaceted molecule with pleiotropic effects in quite divergent cell types, including cancer cells. Many solid tumors exhibit derangements of the nerve growth factor and its receptors, including the tropomyosin receptor kinase A. This receptor is expressed in triple-negative breast cancer, although its role in the pathogenesis and aggressiveness of this disease is still under investigation. We now report that triple-negative breast cancer-derived MDA-MB-231 and MDA-MB-453 cells express appreciable levels of tropomyosin receptor kinase A and release a biologically active nerve growth factor. Activation of tropomyosin receptor kinase by nerve growth factor treatment positively affects the migration, invasion, and proliferation of triple-negative breast cancer cells. An increase in the size of triple-negative breast cancer cell spheroids is also detected. This latter effect might occur through the nerve growth factor-induced release of matrix metalloproteinase 9, which contributes to the reorganization of the extracellular matrix and cell invasiveness. The tropomyosin receptor kinase A inhibitor GW441756 reverses all these responses. Co-immunoprecipitation experiments in both cell lines show that nerve growth factor triggers the assembly of the TrkA/β1-integrin/FAK/Src complex, thereby activating several downstream effectors. GW441756 prevents the complex assembly induced by nerve growth factor as well as the activation of its dependent signaling. Pharmacological inhibition of the tyrosine kinases Src and FAK (focal adhesion kinase), together with the silencing of β1-integrin, shows that the tyrosine kinases impinge on both proliferation and motility, while β1-integrin is needed for motility induced by nerve growth factor in triple-negative breast cancer cells. The present data support the key role of the nerve growth factor/tropomyosin receptor kinase A pathway in triple-negative breast cancer and offer new hints in the diagnostic and therapeutic management of patients.

## Introduction

Despite the significant progress in diagnosis and treatment, breast cancer (BC) still represents a global challenge. Additionally, a specific BC subtype lacking estrogen or progesterone receptor (ER or PR, respectively) and not exhibiting HER2 overexpression/amplification has attracted the attention of oncologists. This subtype is commonly defined as triple-negative breast cancer (TNBC) and accounts for approximately 10–20% of all BCs. TNBC can be considered as a heterogeneous disease, often associated with a worse prognosis. Specific treatments for this cancer are still lacking, and chemotherapy represents the main therapeutic option in the early as well as advanced stages of the disease (Marra et al., 2020). This scenario has been made even more intricate by the discovery of a specific TNBC subtype, characterized by the expression of the androgen receptor (AR) (Lehmann et al., 2011). These findings, together with the identification of various “druggable” biomarkers (e.g., the signaling effectors of the PI3-K- or Ras-dependent pathways), have paved the way for the use of AR- or PI3-K- or MEK-targeted agents in monotherapy or combinatorial therapy for TNBC. TNBC patients, however, often exhibit intrinsic resistance to therapies or acquire drug resistance (Bianchini et al., 2016). The identification of new predictive response biomarkers and therapeutics is needed for the clinical management of TNBC patients.

The neurotrophin β-nerve growth factor (β-NGF, referred to as NGF hereafter) activates two structurally unrelated receptors: the p75 neurotrophin receptor (p75^*NTR*^, also called NGF receptor), which binds all the neurotrophins, and the receptor tyrosine kinase A (TrkA), which shows high-affinity binding to NGF (Huang and Reichardt, 2003). The last years have seen intense investigations on the role of NGF and its receptors in human cancers. As such, many compounds targeting Trkreceptors have been designed and studied for their effects in cultured cancer cells as well as mouse models (Vaishnavi et al., 2015; Drilon et al., 2018; Konicek et al., 2018; Meldolesi, 2018; Smith et al., 2018).

NGF and its receptors play a role in BC. TrkA levels have a prognostic value in BC patients (Descamps et al., 2001a), and secretory BCs are driven by oncogenic *ETV6*–*NTRK3* gene fusions (Lee et al., 2014). NGF signaling fosters the survival and proliferation of BC cells (Descamps et al., 1998, 2001b), and the anti-estrogen tamoxifen inhibits this effect (Chiarenza et al., 2001). These findings support a role for NGF signaling in the pathogenesis and progression of BC. Scant evidence, however, has been so far reported on the role of NGF signaling in TNBC.

In this manuscript, we investigated the role of NGF signaling on the aggressiveness of two TNBC cell lines and the resulting effects of NGF signaling inhibition in these cells. We have employed the MDA-MB-231 and MDA-MB-453 cell lines, which represent the mesenchyme and luminal phenotypes of TNBC-derived cells, respectively (Cailleau et al., 1978; Doane et al., 2006). Albeit at different extents, both cell lines express TrkA and secrete significant amounts of NGF, whose biological activity is neutralized by a specific anti-NGF antibody. Challenging of TNBC cells with NGF activates TrkA and its dependent downstream signaling. Such activation results in mitogenesis, motility, invasion, and a significant increase in the TNBC cell spheroid size. Molecular analysis indicates that NGF challenge triggers the assembly of the TrkA/β1-integrin/FAK/Src complex in TNBC cells. Pharmacological inhibition of TrkA prevents the TrkA/β1-integrin/FAK/Src complex assembly and reverses the mitogenesis and motility in NGF-treated TNBC cells. Similar data were detected using Src or FAK tyrosine kinase inhibitors, while somatic knockdown of β1-integrin only impairs the NGF-elicited motility in TNBC cells.

Taken together, our results dissect the molecular mechanism of NGF action in TNBC cells and indicate that pharmacological inhibitors against TrkA and humanized anti-NGF antibodies might profitably be used as therapeutic tools in TNBC.

## Materials and Methods

### Chemicals

NGF (Millipore, Burlington, MA, United States) and GW441756 (Selleckchem, Munich, Germany) were used at 100 ng/ml and 1 μM, respectively, throughout the manuscript. The Src tyrosine kinase inhibitor SU6656 (Cayman Chemical, Ann Arbor, MI, United States) was used at 5 μM. The FAK inhibitor defactinib (VS-6063, Selleckchem) was used at 10 μM.

### Cell Cultures

The human TNBC-derived cells MDA-MB-231 and MDA-MB-453 and the human prostate cancer-derived C42-B cells were from the Cell Bank Interlab Cell Line Collection (ICLC; Genoa, Italy). Rat pheocromocytoma-derived PC12 cells were from the European Collection of Authenticated Cell Culture (ECACC; Public Health England, London, United Kingdom). The suppliers authenticated the cell lines for DNA profiles using short tandem repeat (STR) analysis. Cells were maintained at 37°C in a humidified 5% CO_2_ atmosphere. Unless otherwise stated, the media and supplements were from Gibco (Thermo Fisher Scientific, Waltham, MA, United States). MDA-MB-231 cells were cultured in phenol red Dulbecco’s modified Eagle’s medium (DMEM) containing 10% fetal bovine serum (FBS), 100 U/ml penicillin, 100 U/ml streptomycin, and 2 mM glutamine. MDA-MB-453 cells were grown in phenol red DMEM/F12 containing 10% FBS, 100 U/ml penicillin, 100 U/ml streptomycin, 2 mM glutamine, and 10 μg/ml insulin (Roche, Basel, Switzerland). Twenty-four hours before stimulation, growing MDA-MB-231 and MDA-MB-453 cells at 70% confluence were made quiescent using phenol red-free DMEM containing 0.1% charcoal-stripped serum (CSS), 100 U/ml penicillin, and 100 U/ml streptomycin. PC12 cells were cultured in Corning plates using F12K medium (ATCC) supplemented with 2.5% FBS, 15% horse serum, streptomycin at 100 μg/ml, and penicillin at 100 U/ml. The cells were made quiescent using DMEM containing 0.1% FBS, antibiotics, and L-glutamine (Gibco) at 2 mM. C42-B cells were cultured as reported (Di Donato et al., 2019). All the cell lines were routinely monitored for mycoplasma contamination. Cell quiescence was evaluated by fluorescence-activated cell sorting (FACS) analysis, as reported (Castoria et al., 2014). It indicates that a large number (almost 85%) of TNBC cells were in G0/G1 (not shown). Cell quiescence was also monitored by 5-bromo-2′-deoxyuridine (BrdU) incorporation analysis, as reported in the subsequent section.

### Phase-Contrast Microscopy, Immunofluorescence, DNA Synthesis, WST-1, and Cyto 3D Live–Dead Assays

PC12 (3 × 10^4^) cells were made quiescent for 24 h and embedded in 250 μl of phenol red-free growth factor-reduced Matrigel (10 mg/ml; BD Biosciences, San Jose, CA, United States). Conditioned medium (CM) derived by TNBC cells unchallenged or challenged for 10 days with anti-NGF neutralizing antibody (1,600 pg/ml) was collected and added to PC12 cells. After 6 days, different fields were analyzed using a Leica DMIRB (Leica, Wetzlar, Germany) microscope equipped with C-Plan ×40 or HCX PL Fluotar ×63 objective (Leica). Images were captured using a DFC 450C camera (Leica). TNBC cells on coverslips were made quiescent and after 72 h were rinsed with phosphate-buffered saline (PBS), fixed for 10 min with paraformaldehyde (4%, *w*/*v*, in PBS; Merck, Saint Louis, MO, United States), permeabilized for 10 min with Tween (0.1%, *v*/*v*, in PBS; Bio-Rad, Hercules, CA, United States), and incubated for 1 h with PBS containing FBS (1%, vol/vol). Cells on coverslips were then incubated with the anti-NGF (1:100, ab6199; Abcam, Cambridge, United Kingdom) antibody overnight at 4°C. After extensive washings in PBS, the coverslips were incubated for 1 h at 37°C with diluted (1:200 in PBS containing 0.01% BSA) fluorescein-conjugated AffiniPure anti-rabbit immunoglobulin G (IgG) (Jackson ImmunoResearch Laboratories, West Grove, PA, United States). When indicated in the figures, the nuclei were stained for 5 min with Hoechst 33258 (1 μg/ml; Merck) and the plasma membrane for 10 min with red fluorescent Alexa Fluor^®^ 594 wheat germ agglutinin (WGA; 5 μg/ml) (Molecular Probes, Invitrogen Ltd., Paisley, United Kingdom). The number of cells positive for NGF (NGF-positive cells) was determined using the formula: percentage of NGF-positive cells = (No. of NGF or pro-NGF-positive cells/No. of total cells) × 100. DNA synthesis was analyzed by BrdU incorporation. To this end, quiescent cells on coverslips were left unchallenged or challenged with NGF in the absence or presence of the indicated compounds for 18 h. After *in vivo* pulse with 100 μM BrdU (Sigma-Aldrich, St. Louis, MO, United States), BrdU incorporation into the newly synthesized DNA was analyzed as reported (Pagano et al., 2004) using a DMLB (Leica, Wetzlar, Germany) fluorescent microscope equipped with HCX PL Apo ×63 oil and HCX PL Fluotar ×100 oil objectives. Images were captured using a DC480 camera (Leica) and acquired using the Leica Suite software. BrdU incorporation was calculated using the formula: percentage of BrdU-positive cells = (No. of BrdU-positive cells/No. of total cells) × 100. Only PC12 cells that, under basal conditions, incorporated <10% BrdU were used in the indicated experiments. WST-1 reagent (Roche) was used to analyze TNBC cell proliferation, as reported (Di Donato et al., 2019). The resulting values were expressed as the fold increase over the basal level. The Cyto3D live–dead assay kit (TheWell Bioscience, North Brunswick, NJ, United States) was used to detect apoptotic TNBC cells. The kit was used according to the manufacturer’s instructions. Dead cells were visualized by using a DMIRB inverted microscope (Leica) equipped with N-Plan ×10 or HCX PL Fluotar ×40 objective (Leica), and the percentage of dead cells was determined using the formula: [No. of propidium iodide (PI)-positive cells/No. of acridine orange (AO)-positive cells] × 100.

### Enzyme-Linked Immunosorbent Assay

**FIGURE 1:**
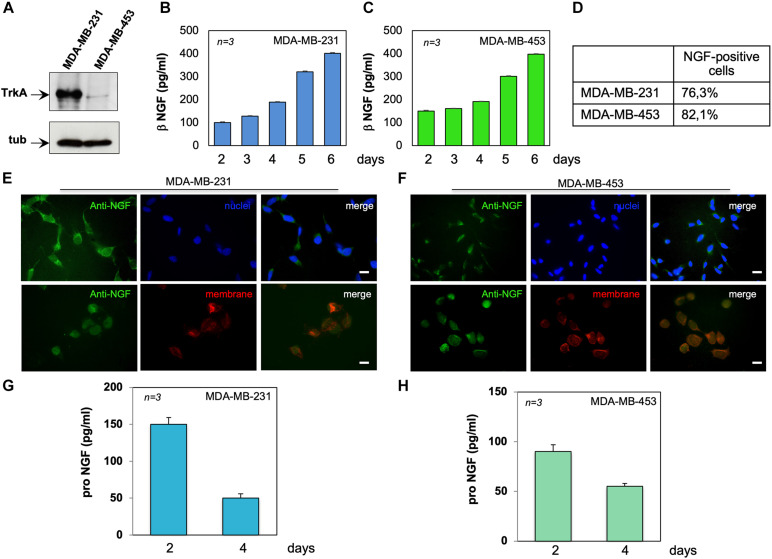
MDA-MB-231 and MDA-MB-453 cells express tyrosine kinase A (TrkA) and release active neurotrophin β-nerve growth factor (NGF). **(A)** Lysates from the indicated cell lines were prepared and the lysate proteins analyzed by Western blotting (WB) using the antibodies against the indicated proteins. **(B,C)** MDA-MB-231 **(B)** and MDA-MB-453 **(C)** cells were made quiescent. Conditioned media (CM) were collected at the indicated times and the amount of β-NGF (in picograms per milliliter) was analyzed. Quiescent MDA-MB-231 and MDA-MB-453 cells on coverslips were stained for NGF. **(D)** Quantification of the cells positive for NGF immunostaining. **(E,F)** Representative immunofluorescence (IF) images from three different experiments (each in duplicate) were captured for MDA-MB-231 cells **(E)** and MDA-MB-453 **(F)** cells. For each experiment, at least 300 cells were scored. NGF (*green*), nuclei (*blue*), and the plasma membrane (*red*) were stained. *Right panels* show the merged images. *Scale bar*, 10 μM. **(G,H)** CM from MDA-MB-231 **(G)** and MDA-MB-453 **(H)** cells were collected at the indicated times and the amount of pro-NGF (in picograms per milliliter) was analyzed by ELISA. Means and SEMs are shown. *n* represents the number of experiments.

**FIGURE 2:**
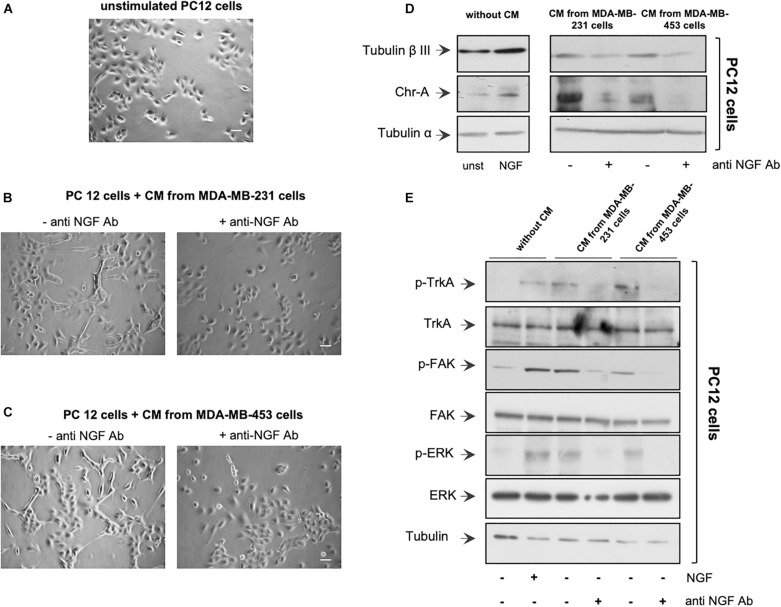
Neurotrophin β-nerve growth factor (NGF) secreted by triple-negative breast cancer (TNBC) cells is biologically active. **(A,B)** PC12 cells were embedded in Matrigel and unchallenged **(A)** or challenged with conditioned media (CM) from MDA-MB-231 **(B)** or from MDA-MB-453 **(C)** cells (*left panels*) in the absence or presence of anti-NGF neutralizing antibody (anti-NGF; *right panels*) for 6 days. Phase-contrast images are representative of three different experiments, each in duplicate. *Scale bar*, 10 μM. **(D,E)** PC12 cells were unstimulated or stimulated for 72 h with 100 ng/ml NGF or CM from MDA-MB-231 and MDA-MB-453 cells unchallenged or challenged with anti-NGF. Lysate proteins from PC12 cells were analyzed by Western blot (WB) using the antibodies against the indicated proteins. WB are representative of three different experiments.

TNBC cells (8 × 10^4^ in six-well plates in Figures 1B,C,G,H; 55 × 10^4^ in 100-mm plates in Supplementary Figure 1E and the corresponding experiments in Figure 2) were made quiescent. The cell culture media were collected at the times indicated in the figures and the corresponding legends. Enzyme-linked immunosorbent assay (ELISA) kits for β-NGF (EHNGF; Thermo Fisher Scientific) and pro-NGF (MBS706083; MyBioSource, San Diego, CA, United States) were used for the quantitative determination of β-NGF and pro-NGF in the cell culture media. The resulting data were analyzed using the curve-fitting statistical software GraphPad Prism.

### Wound Scratch Assay, Boyden Chamber Migration Assay, and Invasion Assay

In the wound scratch analysis, 1.8 × 10^5^ cells were seeded in a 24-well plate. The cells were made quiescent, wounded using 10-μl sterile pipette tips, and left unstimulated or stimulated for 12 h with NGF in the absence or presence of the indicated compounds. To avoid cell proliferation, cytosine arabinoside (Sigma-Aldrich) at 50 μM (final concentration) was included in the cell medium. Different fields were analyzed using a DMIRB inverted microscope (Leica) equipped with N-Plan ×10 objective (Leica), as reported (Giovannelli et al., 2019). Phase-contrast images were captured using a DFC 450C camera (Leica) and acquired using the Application Suite software (Leica). Images are representative of at least three different experiments. The wound gap was calculated using ImageJ software and expressed as the percentage of decrease in the wound area. Migration and invasion assays were done as reported (Giovannelli et al., 2019) using quiescent MDA-MB-231 or MDA-MB-453 cells in collagen (for migration assay) or Matrigel (for invasion assay) pre-coated Boyden chambers with 8 μm polycarbonate membrane (Corning, Corning, NY, United States). The indicated compounds were added and cytosine arabinoside (Sigma-Aldrich) was included (at 50 μM final concentration) in the cell medium. The cells were allowed to migrate or invade for 7 or 18 h, respectively. Migrating or invading cells were finally stained with Hoechst 33258 and scored (Giovannelli et al., 2019).

### Gelatine Metalloproteinase Zymography

Zymography assay was done using MDA-MB-231 cells at 80% of confluence. The cells were made quiescent, left in serum-free media, and then unstimulated or stimulated for 30 h with NGF in the absence or presence of GW441756. CM was collected and centrifuged, while the cells were detached by trypsin and counted. CM was normalized to 1 × 10^6^ MDA-MB-231 cells and MMP-9 proteolytic activity was assayed in CM as reported (Di Donato et al., 2021). It appeared as a clear band migrating at ≈92 kDa on a blue background.

### 3D Cultures and Spheroid’s Viability by MTT Assay

**FIGURE 3:**
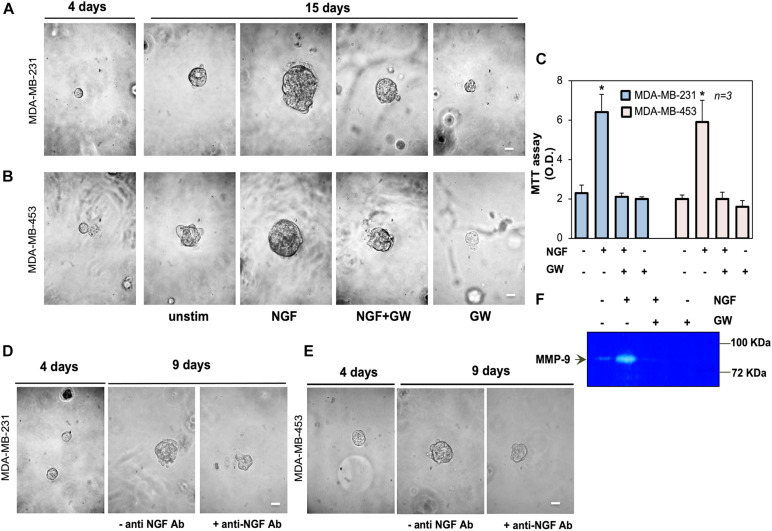
Neurotrophin β-nerve growth factor (NGF) challenge increases the size of triple-negative breast cancer (TNBC) cell spheroids and the MMP-9 release. **(A,B)** MDA-MB-231 **(A)** and MDA-MB-453 **(B)** cells embedded in Matrigel were used. Four days later, representative images were acquired. Cells were left untreated or treated with 100 ng/ml NGF in the absence or presence of GW441756 (GW; 1 μM) for 15 days. Shown are phase-contrast images, representative of three different experiments, captured on the 15th day. *Scale bar*, 100 μm. **(C)** Spheroid viability was analyzed by the MTT assay after 15 days of treatment. Optical density (OD) was measured at 562 nm and the obtained results are shown in the graph. Means and SEMs are shown. *n* represents the number of experiments. **(D,E)** MDA-MB-231 **(D)** and MDA-MB-453 **(E)** cells were used to generate spheroids, as in **(A,B)**. Spheroids were left untreated for 9 days, avoiding refreshing the medium, in the absence or presence of the anti-NGF antibody (anti-NGF Ab). Shown are phase-contrast images, representative of three different experiments, captured at day 9. *Scale bar*, 100 μm. **(F)** MDA-MB-31 cells were treated for 30 h with 100 ng/mL NGF in the absence or presence of GW441756 (GW; 1 μM). The release of MMP-9 in conditioned media was analyzed by zymography. *p < 0.05 for the indicated experimental points vs. the corresponding untreated controls.

Spheroids were generated as reported (Di Donato et al., 2021). MDA-MB-231 and MDA-MB-453 cells (3 × 10^4^) were mixed in each well with 250 μl of phenol red-free growth factor-reduced Matrigel (10 mg/ml; BD Biosciences) and 50 μl of spheroid plating medium. It was made using phenol red-free DMEM/F12 medium containing 7% CSS, 100 U/ml penicillin, 100 U/ml streptomycin, GlutaMAX 100× (Gibco), 10 mM HEPES, 1 M nicotinamide (Merck), 500 mM *N*-acetylcysteine (Sigma-Aldrich), and 10 μM Y-27632 (Merck). After 3 days, the spheroid plating medium was replaced with a similar medium in the absence of *N*-acetylcysteine and Y-27632. On day 4, the spheroids were untreated or treated with the indicated compounds. Unless otherwise stated, the medium was changed every 2 days. In Figures 3D,E, the media were not changed until the 9th day. Different fields were analyzed using Leica DMIRB (Leica) microscope equipped with C-Plan ×40 objective (Leica) and phase-contrast images were acquired using a DFC 450C camera (Leica). The relative spheroid size was calculated using the Application Suite software (Leica) and expressed as a fold increase over the basal spheroid size, which was measured on the 3rd day. After 15 days, spheroid viability was assessed with 3-(4,5-dimethylthiazol-2-yl)-2,5-diphenyltetrazolium bromide (MTT; Sigma-Aldrich). Briefly, MDA-MB-231 and MDA-MB-453 spheroids were incubated with a 2% (*w*/*v*) SDS solution to solubilize the Matrigel. After 2 h at 37°C, the MTT solution (final concentration of 500 μg/ml) was added to the spheroids at 37°C and 5% CO_2_. Two hours later, DMSO (100 μl) was added and the mixture was incubated for 1 h at 37°C. The optical density (OD) from duplicate samples was measured at 562 nm using an EnSpire plate reader (PerkinElmer, Waltham, MA, United States).

### Transfections and siRNA Experiments

Growing MDA-MB231 cells were transfected using Lipofectamine 2000 (Invitrogen). For β1-integrin small interfering RNA (siRNA), a pool of three to five target-specific 19–25 siRNAs (Santa Cruz, Dallas, TX, United States) was used. Non-targeting siRNA [control (ctrl) siRNA], containing a scrambled sequence, was from Santa Cruz. The cells were co-transfected with 2 μg eGFP-cDNA (Lonza, Milan, Italy) to help in the identification of transfected cells. After 6 h, transfected cells were made quiescent for 24 h and then used.

### Lysates, Immunoprecipitation, Co-immunoprecipitation, and Western Blot

All these were done as reported (Di Donato et al., 2019). The following reagents were used: mouse monoclonal anti-p75 (B-1, sc-271708; Santa Cruz), anti-FAK (610088; BD Transduction Laboratories), or anti P-Tyr 397 FAK (611722; BD Transduction Laboratories); anti-Src (sc-8056; Santa Cruz), anti-p42 extracellular signal-regulated kinase (ERK) (sc-1647; Santa Cruz), or anti p44 and p42 P-ERK (sc-7383; Santa Cruz); anti-tubulin (T5168; Sigma-Aldrich) antibodies; and rabbit polyclonal anti-TrkA (06-574; Millipore), P-Tyr490 TrkA (#9141; Cell Signaling, Danvers, MA, United States), β1-integrin (Ab1952; Millipore), P-Thr 638/641 PKCα/β II (#9375; Cell Signaling), and P-Ser 643/676 PKCδ/θ (#9376; Cell Signaling) antibodies. The ECL system (GE Healthcare, Chicago, IL, United States) was used to reveal immunoreactive proteins.

### Statistical Analysis

Results are expressed as the mean ± SEM of at least three independent experiments. Two-tailed unpaired Student *t*-tests and one-way or two-way ANOVA (Bonferroni’s *post-hoc* test) were used, where appropriate.

## Results

### TNBC Cells Express TrkA and Release Biologically Active NGF

The expression of the NGF receptor TrkA was analyzed by Western blot (WB) technique in lysate proteins from MDA-MB-231 and MDA-MB-453 cells. The anti-TrkA antibody revealed an immunoreactive band migrating at 140 kDa, the expected molecular weight of TrkA. The amount of immunoreactive bands in MDA-MB-231 cells was much higher than that observed in MDA-MB-453 cells (Figure 1A), and similar results were obtained from three different experiments (Supplementary Figure 1). Despite significant levels of the neurotrophin receptor family member p75 have been recently detected in a lung metastatic clone from modified MDA-MB231 cells (Wu et al., 2021), we did not observe in the WB analysis robust levels of p75 (Supplementary Figures 2A,B), regardless of the TNBC cell line. These apparent discrepancies might be due to the quite different clones of TNBC cells used. Nevertheless, other critical factors, such as the stromal microenvironment as well as the signal- and context-dependent interactions between BC metastatic cells and the lung cellular components, might influence the re-expression of p75 in the lung metastatic clone of MDA-MB-231.

Since BC cells release NGF (Adriaenssens et al., 2008), we measured the NGF content in CM from MDA-MB-231 (Figure 1B) or MDA-MB-453 (Figure 1C) cells. Data from ELISA in Figures 1B,C show that both TNBC cell lines release appreciable amounts of NGF, already after 2 days to reach elevated levels (about 400 pg/ml) after 6 days of cell culture. Immunofluorescence (IF) quantification (Figure 1D) and images (Figures 1E,F) reveal that almost 77% of MDA-MB-231 (Figures 1D,E) and 82% of MDA-MB-453 (Figures 1D,F) cells were positive for NGF immunostaining. In both cell lines, NGF staining was prevalently seen in the extranuclear compartment (upper panels in Figures 1E,F), close to the plasma membranes (red; lower panels in Figures 1E,F), and the specificity of the IF approach was confirmed by the absence of fluorescence in the control staining, obtained from the secondary antibody alone (Supplementary Figures 2C,D).

Because of previous findings on the role of pro-NGF in BC aggressiveness (Lévêque et al., 2019), we also analyzed the release of pro-NGF from TNBC cells. After 2 days of culture, MDA-MB-231 (Figure 1G) and MDA-MB-453 (Figure 1H) cells secreted almost 150 and 100 pg/ml of pro-NGF, respectively. Such amounts decreased over the time, to reach almost undetectable levels (<50 pg/ml) after 4 days of culture. Thus, a low amount of pro-NGF is released by TNBC cells in our setting, suggesting that NGF, rather than pro-NGF, might sustain the aggressiveness of TNBC cells through an autocrine loop in our conditions.

We then verified whether the NGF secreted by TNBC cells is biologically active. CM from a robust number of TNBC cells (see also “Materials and Methods”) was collected after 10 days of cell culture. About 4,867 and 6,923 pg/ml of NGF were found in CM collected from MDA-MB-231 and MDA-MB-453 cells, respectively (Supplementary Figure 2E). Therefore, we verified whether CM from MDA-MB-231 or the MDA-MB-453 cell line induces a neuronal phenotype in rat adrenal pheochromocytoma PC12 cells (Greene and Tischler, 1976), which undergo differentiation on NGF stimulation (Marshall, 1995). The cells were embedded in Matrigel to simulate the complexity of the extracellular matrix (ECM), and the effect of CM addition was evaluated after 6 days. Contrast-phase images show that CM from MDA-MB-231 (Figure 2B) or MDA-MB-453 (Figure 2C) cells induced the acquisition of a stellate shape and the development of neurites (left panels) in PC12 cells when compared with the unstimulated PC12 cells (Figure 2A). Notably, when the neutralizing anti-NGF antibody was added to CM from TNBC cells, PC12 cells remained in a round and undifferentiated shape (right panels). Since we have observed that NGF treatment of PC12 cells upregulates β-tubulin III and chromogranin A (Chr-A) expressions (Di Donato et al., 2015), we also evaluated the levels of these proteins. In the absence of CM, challenging of PC12 cells with NGF upregulated the β-tubulin III and Chr-A levels after 3 days (left section in Figure 2D). A significant expression of β-tubulin III and a robust bulk of Chr-A were detected by adding to PC12 cells the CM derived from TNBC cells. Here, again, CM treatment with the anti-NGF neutralizing antibody resulted in a decrease in the levels of both β-tubulin III and Chr-A (right section in Figure 2D), with a more robust effect detectable on this latter marker. Thus, NGF contained in the CM from TNBC cells seems to be responsible for the observed effects in PC12 cells. To strengthen this finding, we investigated the effect of CM from TNBC cells on the activation of various effectors involved in NGF signaling. In the absence of CM, NGF challenging of PC12 cells increased the Tyr-490 TrkA phosphorylation as well as FAK and p44-p42 ERK activation. In the presence of CM, we still observed these effects in PC12 cell lysate proteins. The addition of neutralizing anti-NGF antibody to CM inhibited the activation of various NGF signaling components (Figure 2E).

Taken together, the data in Figures 1, 2 and Supplementary Figure 2 indicate that TNBC cells express TrkA and release biologically active NGF. As such, an autocrine loop might sustain the proliferation and aggressiveness of TNBC cells.

### NGF Treatment Increases the Size of TNBC Cell Spheroids

We next established a three-dimensional culture system in MDA-MB-231 and MDA-MB-453 cells using growth factor-reduced Matrigel. This condition allows the formation of spheroids after 4 days of culture, as shown by the phase-contrast microscopy images in Figures 3A,B. On day 4 of culture, the spheroids were left untreated or treated with NGF in the absence or presence of the TrkA specific inhibitor GW441756 (Wood et al., 2004). The cell medium was refreshed every 2 days and changes in the spheroid size were monitored for 15 days. Phase-contrast microscopy images were captured (Figures 3A,B) and the quantification of data was also done (Supplementary Figure 1F). After 15 days, NGF increased by about 11- and 10-fold the sizes of the MDA-MB-231 and MDA-MB-453 spheroids, respectively. GW441756 significantly (*p* < 0.05 in Figures 3A,B and Supplementary Figure 2F) reduced the NGF effect, leaving almost unaltered the spheroid size when used alone, as a control. We also analyzed the effect of NGF on the spheroid’s viability with the MTT assay. Figure 3C shows that NGF increased by about sixfold the viability of spheroids from both TNBC cell lines and that GW441756 decreased such effect. The inhibitor did not modify the spheroid viability when used alone, as a control. Since the NGF-mediated autocrine loop may be already involved in the “basal” conditions, we generated TNBC cell spheroids avoiding refreshing the medium. On day 4 of culture, the spheroids were left untreated in the absence or presence of the anti-NGF antibody. Changes in the spheroid size were then monitored for 9 days and phase-contrast microscopy images were captured. The images in Figures 3D,E, together with the quantification of data (Supplementary Figure 2G), show that the sizes of the TNBC cell spheroids increased by almost fourfold. The addition of the anti-NGF antibody reduced such effect. Taken together, these findings demonstrate for the first time a role for NGF and TrkA activation in fueling the size of TNBC-derived spheroids.

The release of matrix metalloproteinases (MMPs) is crucial for ECM remodeling, cell invasion, and tumor progression (Bonnans et al., 2014; Chatterjee et al., 2018; Yuzhalin et al., 2018). Since NGF induces MMP-9 release (Khan et al., 2002), we analyzed the effect of NGF on MMP-9 release by MDA-MB-231 cells. NGF robustly increased the secretion of MMP-9, as assessed using the results from zymography in Figure 3F. GW441756 inhibited this effect. Untreated cells, which were maintained in serum-free medium for 30 h, released a low MMP-9 amount, likely because of the scant quantity (<95 pg/ml) of NGF secreted by MDA-MB231 cells at that time (not shown). By affecting such a basal condition, GW441756 slightly perturbed the MMP-9 release, when used alone (Figure 3F). The data in panel F support a role for the NGF-induced MMP-9 release in ECM remodeling and the consequent increase in TNBC cell spheroid size.

### NGF Treatment Induces DNA Synthesis and Proliferation in TNBC Cells

**FIGURE 4:**
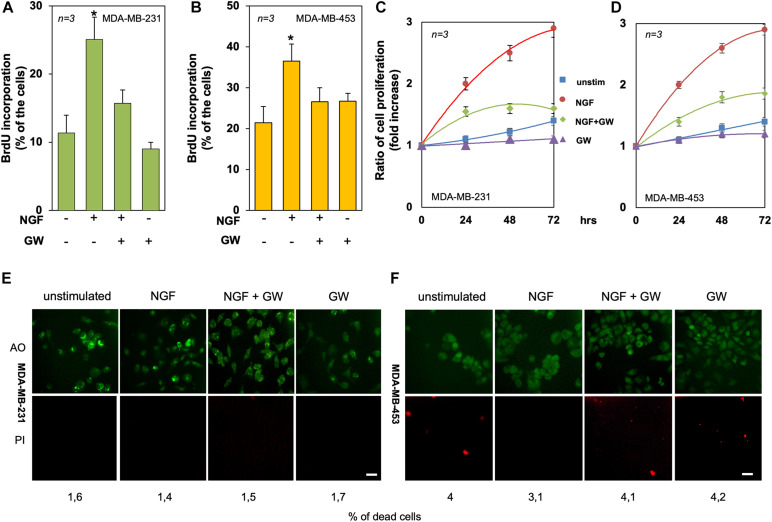
Tyrosine kinase A (TrkA) mediates the neurotrophin β-nerve growth factor (NGF) mitogenic effect in triple-negative breast cancer (TNBC) cells. **(A,B)** Quiescent MDA-MB-231 **(A)** and MDA-MB-453 **(B)** cells were left untreated or treated for 18 h with the indicated compounds. Cells were pulsed *in vivo* with 100 μM BrdU and its incorporation into DNA was analyzed by immunofluorescence (IF) and expressed as the percentage of total cells. **(C,D)** Quiescent MDA-MB-231 **(C)** and MDA-MB-453 **(D)** cells were left untreated or treated for 24, 48, and 72 h with the indicated compounds. Cell proliferation was assayed using the WST-1 reagent. Graphs represent the ratios of proliferation, which was expressed as the fold increase over the basal absorbance. NGF stimulation induced a significant (*p* < 0.05) increase in cell proliferation as compared with the untreated cells. **(E,F)** MDA-MB-231 **(E)** and MDA-MB-453 **(F)** cells were untreated or treated for 72 h with the indicated compounds and stained with the Cyto3D reagent. Shown are IF images of the total (*green*) and dead (*orange*) cells, representative of three different experiments. *Scale bar*, 10 μm. The percentages of dead cells for each point are indicated in the figure. In **(A–F)**, NGF was used at 100 ng/ml and GW441756 (GW) was used at 1 μM. Means and SEMs are shown. *n* represents the number of experiments. *p < 0.05 for the indicated experimental points *vs*. the corresponding untreated control.

To evaluate the mitogenic effect of NGF in TNBC-derived cell lines, BrdU incorporation and proliferation assays were done. NGF stimulation increased by about 2.5- and 1.8-fold the number of MDA-MB-231 (Figure 4A) and MDA-MB-453 (Figure 4B) cells incorporating BrdU as compared to unstimulated cells. GW441756 impaired the NGF-elicited effect, indicating that TrkA activity is required for this response. The inhibitor did not significantly modify the BrdU incorporation when used alone in both cell lines (Figures 4A,B). The effect of NGF on cell proliferation was further evaluated by the WST-1 assay. NGF treatment stimulated the proliferation in both TNBC cell lines, with an effect already evident after 24 h, while GW441756 inhibited the NGF-elicited effect, which persists until 72 h of treatment (Figures 4C,D). Since the neurotrophin receptor TrkA drives the death of developing neurons *in vitro* and *in vivo* (Nikoletopoulou et al., 2010), we used a live–dead assay to address this issue. Irrespective of cell treatment, a negligible effect on the number of TNBC dead cells was detectable only after 3 days (Figures 4E,F).

In summary, TrkA activation by NGF drives the DNA synthesis and proliferation in both TNBC cell lines.

### NGF Treatment Induces Migration and Invasion in TNBC Cells

**FIGURE 5:**
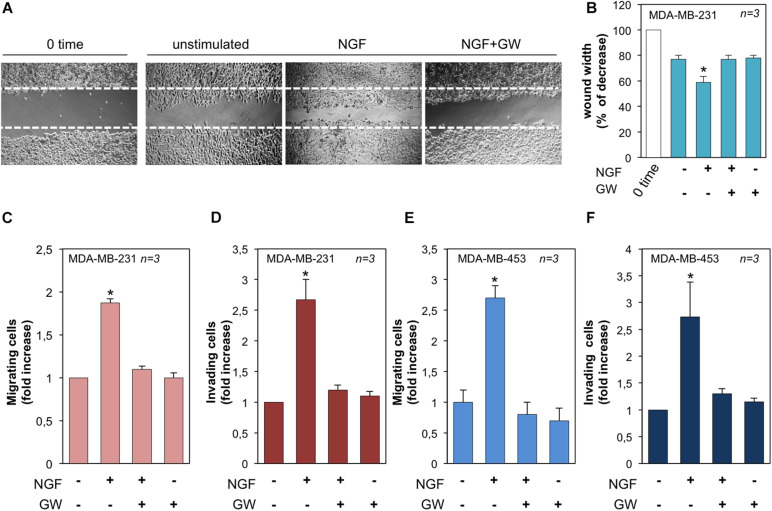
Neurotrophin β-nerve growth factor (NGF) challenge induces migration and invasiveness in triple-negative breast cancer (TNBC) cells. **(A)** Quiescent MDA-MB-231 cells were wounded and left unstimulated or stimulated with NGF for 18 h in the absence or presence of GW441756 (GW). Phase-contrast images are representative of three different experiments, each in duplicate. **(B)** Wound area calculated using Leica Suite software. Data are presented as the percentage of decrease in wound width over the control cells, analyzed at time 0. **(C–F)** Quiescent MDA-MB-231 **(C)** and MDA-MB-453 **(E)** cells were used for migration assays in Boyden chambers pre-coated with collagen. Quiescent MDA-MB-231 **(D)** and MDA-MB-453 **(F)** cells were used for invasion assays in Boyden chambers pre-coated with Matrigel. The indicated compounds were added to the upper and the lower chambers and the cells were counted as reported in “Materials and Methods.” Results from three different experiments were collected and expressed as fold increase. In **(B–F)**, means and SEMs are shown. *n* represents the number of experiments. *p < 0.05 for the indicated experimental points *vs*. the corresponding untreated control.

We next evaluated the effect of NGF on the motility and invasion of TNBC cells. In a first attempt, MDA-MB-231 cells were wounded and allowed to migrate in the absence or presence of the indicated compounds. Phase-contrast images from the wound scratch assay show that a significant number of cells migrated in the wound area upon NGF treatment, while GW441756 inhibited the NGF-induced effect. Images captured at time 0 or from untreated cells were also captured and presented for comparison (Figure 5A). Data from three different experiments are graphically shown in Figure 5B. They indicate that the wound width was significantly (*p* < 0.05) reduced in cells treated with NGF as compared with the control untreated cells. GW441756 reverted the effect elicited by NGF while exhibiting a negligible effect when used alone. We avoided the wound scratch assay in MDA-MB-453 cells since they are semi-adherent and not rightly available in this approach (Giovannelli et al., 2019). Finally, we studied the NGF effect on the migration and invasiveness of TNBC cells by using collagen- and Matrigel-coated Boyden chambers. NGF increased by ∼2- and 2.7-fold the number of migrating (Figure 5C) or invading (Figure 5D) MDA-MB-231 cells, respectively. The latter results on cell invasion are consistent with the finding that MDA-MB-231 cells release MMP-9 on NGF treatment (see Figure 3). NGF also increased by ∼2.6- and 2.7-fold the number of migrating (Figure 5E) or invading (Figure 5F) MDA-MB-453 cells, respectively. Throughout this set of experiments, GW441756 inhibited the NGF-induced effects while leaving almost unaffected the migration or invasion of TNBC cells when used alone.

### NGF Treatment Triggers the TrkA/FAK/β1-Integrin/Src Complex Assembly and Activates the TrkA-Dependent Signaling Network

**FIGURE 6:**
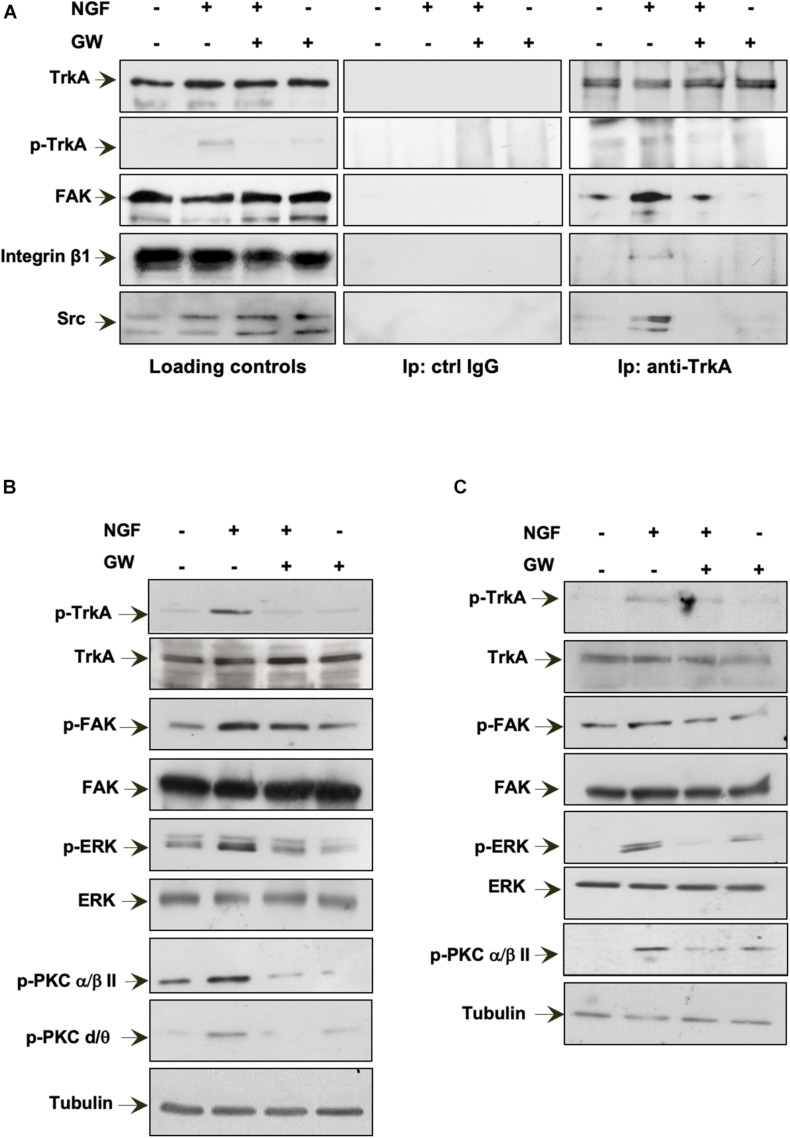
Neurotrophin β-nerve growth factor (NGF) challenge induces the TrkA/FAK/β1-integrin/Src complex assembly and the NGF-dependent signaling activation. **(A,B)** Quiescent MDA-MB-231 cells were left unchallenged or challenged for 15 min with NGF in the absence or presence of GW441756 (GW). In **(A)**, lysate proteins were immunoprecipitated using the anti-TrkA antibody (anti-TrkA; *right panels*) or control immunoglobulin G (ctrl IgG; *middle panels*). Loading controls are shown in *left panels*. Western blot (WB) with the indicated antibodies was done to detect proteins in the immunocomplex. In **(B)**, lysate proteins were analyzed using the antibodies against the indicated proteins. **(C)** Quiescent MDA-MB-453 cells were left unchallenged or challenged for 15 min with NGF in the absence or presence of GW. Lysate proteins were analyzed using the antibodies against the indicated proteins. The filter was stripped and re-probed using anti-tubulin antibody as a loading control. All results are representative of three different experiments. *p-TrkA*, Tyr 490-p-TrkA; *p-FAK*, Tyr 397-p-FAK; *p-ERK*, p44 and p42 ERK; *p-PKC* α/β*II*, Thr 638/41-p-PKC α/βII; *p-PKC* δ/θ, Ser643/676.

In a preliminary time course experiment in MDA-MB-231 cells challenged for different times (from 5 to 30 min) with NGF, we detected a robust co-immunoprecipitation (Co-IP) of TrkA with FAK at 15 min of cell treatment (not shown). Therefore, we selected this time point in the subsequent analysis. MDA-MB-231 cells were then challenged for 15 min with NGF in the absence or presence of GW441756 and the lysates immunoprecipitated with anti-TrkA antibodies. The WB analysis of the Co-IP proteins shows that TrkA was phosphorylated at Tyr490 within 15 min. Simultaneously, a significant Co-IP of TrkA, FAK, β1-integrin, and Src tyrosine kinase was detected upon NGF stimulation (right panels in Figure 6A). GW441756 perturbed the NGF-induced complex assembly. Similar TrkA amounts were detected in the immunocomplexes regardless of the experimental condition, and the Co-IP approach is specific since no proteins were detected in the lysates immunoprecipitated with the control antibodies (middle panels in Figure 6A). Lastly, WB of lysate proteins with the indicated antibodies shows that similar protein amounts were loaded in our approach (left panels in Figure 6A).

NGF binding to TrkA induces the receptor dimerization and phosphorylation of its tyrosine residues. Once phosphorylated, TrkA provides docking sites for the effector molecules, which in turn recruit and activate several signaling effectors, thus propagating different downstream signaling cascades (Biarc et al., 2013). Expectedly, 15 min of NGF stimulation leads to an increase in Tyr-490 TrkA phosphorylation in MDA-MB-231 cells, and GW441756 abolishes such effect, leaving almost unaffected the TrkA phosphorylation when used alone (Figure 6B). As readout of TrkA phosphorylation, we then analyzed the activation of several downstream effectors involved in NGF signaling. NGF robustly increases the activation of FAK as well as p44-p42 ERK phosphorylation. NGF also triggers protein kinase C (PKC; α/β and δ/θ) phosphorylation. GW441756 reverses all the effects induced by NGF, leaving unaltered the activation state of various signaling effectors when used alone, as a control (Figure 6B).

We next analyzed the NGF signaling activation in MDA-MB-453 cells (Figure 6C). As we detected a significant Tyr-490 TrkA phosphorylation within 15 min of NGF stimulation in MDA-MB-231 cells (not shown), we used this time point for the subsequent analysis. A significant increase in FAK as well as p44-p42 ERK activation was detected in NGF-challenged cells, together with PKC phosphorylation (p-PKC; α/β). GW441756 prevented the effects induced by NGF without affecting the activation state of the signaling effectors when used alone (Figure 6C).

In summary, NGF rapidly induces the activation of TrkA and its consequent complexation with β1-integrin as well as the Src and FAK tyrosine kinases. The assembly of this complex leads to the activation of the downstream NGF signaling pathway in TNBC cells.

### Role of TrkA-Dependent Signaling in Biological Responses Elicited by NGF Treatment in TNBC Cells

**FIGURE 7:**
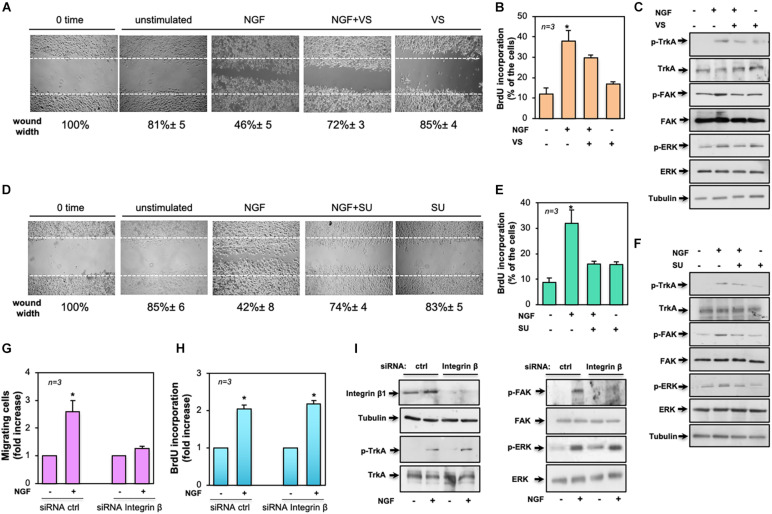
Role of focal adhesion kinase (FAK), Src, and β1-integrin in the biological effects elicited by neurotrophin β-nerve growth factor (NGF) treatment of MDA-MB-231 cells. In **(A–F)**, quiescent MDA-MB-231 cells were used. In **(A–C)**, the FAK inhibitor VS-6063 (VS) was used at 10 μM. In **(D–F)**, the Src inhibitor SU6656 (SU) was used at 5 μM. In **(A–I)**, NGF was used at 100 ng/ml. **(A,D)** Cells were wounded and left unstimulated or stimulated with NGF in the absence or presence of the indicated compounds. Phase-contrast images are representative of three different experiments, each in duplicate. The wound area was calculated using Leica Suite software and data are reported *below each image*. Data are expressed as the percentage of decrease in wound width over the control cells (analyzed at time 0). **(B,E)** Cells were left untreated or treated for 18 h with the indicated compounds. After *in vivo* pulse with 100 μM BrdU, incorporation of BrdU by the cells was analyzed by immunofluorescence (IF) and expressed as the percentage of total cells. **(C,F)** Cells were left unchallenged or challenged for 15 min with NGF in the absence or presence of the indicated compounds. Lysate proteins were analyzed by Western blot (WB) using the antibodies against the indicated proteins. **(G–I)** MDA-MB-231 cells were transfected with β1-integrin or control (ctrl) siRNA in the presence **(G,H)** or absence **(I)** of eGFP-cDNA to help in the identification of transfected cells. **(G)** Quiescent cells were left unstimulated or stimulated with NGF for 7 h and used for the migration assay. Migrated cells were scored using a fluorescent microscope and the data expressed as the relative increase in the number of migrating cells. Data from several independent experiments were collected and analyzed. **(H)** Quiescent cells were left untreated or treated with NGF for 18 h. After *in vivo* pulse with 100 μM BrdU, the DNA synthesis was analyzed by IF and calculated using the formula: percentage of BrdU-positive cells = (No. of transfected BrdU-positive cells/No. of transfected cells) × 100. Data are presented as the fold increase over the basal level. In **(G,H)**, for each plasmid, the data are derived from at least 500 transfected cells. The results of three independent experiments have been averaged. Means and SEM are shown. *n* represents the number of experiments. **(I)** Transfected cells were left unstimulated or stimulated with NGF for 15 min and lysate proteins were analyzed by WB using the antibodies against the indicated proteins. Abbreviations used in **(C,F,E)**: *p-TrkA*, Tyr 490-p-TrkA; *p-FAK*, Tyr 397-p-FAK; *p-ERK*, p44 and p42 ERK. The filter was stripped and re-probed using anti-tubulin antibody as a loading control.

Given the findings that Src and FAK represent key drivers of mitogenesis and invasion in solid cancers (Irby and Yeatman, 2000; Mitra and Schlaepfer, 2006), we investigated their role in NGF-elicited effects. The effect of the FAK inhibitor VS-6063 (Kang et al., 2013) was exploited on motility and mitogenesis induced by NGF in MDA-MB-231 cells. Phase-contrast images from the wound scratch assay show that a significant number of cells migrated in the wound area upon NGF treatment. VS-6063 inhibited the NGF effect. Images captured at time 0 or from unstimulated cells are also shown (Figure 7A). Shown below the images is the corresponding percentage of wound width decrease. FAK activation, however, also plays a role in DNA synthesis, as assessed by the inhibitory effect of VS-6063 on the NGF-induced BrdU incorporation (Figure 7B). VS-6063 did not affect the NGF-induced Tyr-490 Trk phosphorylation, while it inhibited FAK and p44-p42 ERK activation in NGF-treated cells (Figure 7C). Superimposable results in terms of motility (Figure 7D), DNA synthesis (Figure 7E), and signaling activation (Figure 7F) were observed using the Src tyrosine kinase inhibitor SU6656 (Blake et al., 2000). The effect of Src inhibition on NGF-elicited BrdU incorporation was more robust as compared to that observed by FAK inhibition, likely because other members of the Src tyrosine kinase family are engaged by NGF (Dey et al., 2005) to convey its mitogenic signaling, and SU6656 also inhibits these kinases (Blake et al., 2000).

To further address the molecular mechanism underlying NGF signaling in TNBC cells, we silenced β1-integrin, as assessed by the WB analysis in Figure 7I (left panel). The NGF-induced motility (Figure 7G) and DNA synthesis (Figure 7H) of cells were then analyzed. Of note is that β1-integrin knockdown only affected the migratory properties of the NGF-treated cells while leaving unaltered the BrdU incorporation. Again, β1-integrin knockdown did not affect the NGF-induced Tyr-490 TrkA (left panel in Figure 7I) or p44-p42 ERK phosphorylation (right panel in Figure 7I), while it almost completely abolished the NGF-induced FAK activation (right panel in Figure 7I).

Taken together, our findings indicate that NGF-induced TrkA tyrosine phosphorylation controls a plethora of signaling components. In this plot, β1-integrin behaves as a bridge linking FAK to TrkA. Such a connection seems to be required for motility induced by NGF.

## Discussion

Many findings have highlighted the role of TrkA as a driver of cell transformation. They have also suggested that derangement of the NGF circuit is involved in drug resistance, survival, and metastatic spreading of solid tumors (Demir et al., 2016), including the so-called hormone-dependent cancers (Descamps et al., 1998, 2001a, b; George et al., 1998; Pflug and Djakiew, 1998; Sigala et al., 1999; Krygier and Djakiew, 2001, 2002; Miknyoczki et al., 2002; Dollé et al., 2003, 2004; Festuccia et al., 2007; Papatsoris et al., 2007; Anagnostopoulou et al., 2013). Specific targeting of NGF inhibits the proliferation and metastatic events in BC (Adriaenssens et al., 2008). Furthermore, TrkA overexpression has been detected in over 20% of BCs, and it has been linked to their proliferation and spreading (Lagadec et al., 2009; Snowman et al., 2020). Silencing of TrkA enhances chemosensitivity in BC cultured cells and inhibits their spreading in a mouse model (Zhang et al., 2015). These findings point to the role of the NGF/TrkA pathway in BC.

In this study, we have analyzed the role of NGF signaling activation in TNBC cell aggressiveness. MDA-MB-231 and MDA-MB-453 cells both express significant amounts of TrkA and undergo mitogenesis and motility on NGF challenge. Such effects require TrkA activation, as the specific inhibitor GW441756 reverses both the responses. Simultaneously, NGF rapidly triggers the association of TrkA with β1-integrin, FAK, and Src in MDA-MB-231 cells. These effectors are involved in mitogenesis, focal adhesion complex assembly, and migration induced by growth factors, cytokines, neurotrophins, and ECM in various cell types (Roche et al., 1995; Sieg et al., 2000; Bromann et al., 2004). The NGF-triggered TrkA/β1-integrin/FAK/Src complex assembly induces the activation of several downstream effectors, including the p44-p42 ERK and PKCs. GW441756 disrupts the NGF-induced complex assembly and inhibits the TrkA-dependent signaling activation. By this way, the inhibitor reverses the mitogenesis and motility induced by NGF in these cells. Pharmacological inhibition of the Src and FAK tyrosine kinases shows that both the effectors are required for the NGF-elicited proliferation and motility in TNBC cells. Notably, findings from transient knockdown of β1-integrin support the conclusion that its recruitment to the TrkA/FAK/Src complex is needed for the locomotion elicited by NGF in TNBC cells.

Tyrosine kinase A activation is also needed for the NGF-induced increase in TNBC cell spheroid size and viability, as its inhibition by GW441756 results in a significant reduction of these effects. The inhibitory action of GW441756 is consistent with the observed effect on NGF-elicited mitogenesis. However, since NGF treatment also increases the release of MMP-9 in MDA-MB-231 cells, it might be conceived that the NGF-triggered TrkA/β1-integrin/FAK/Src complex assembly constitutes a signaling module relevant to ECM remodeling in TNBC cells. Perturbing the NGF-induced complex assembly and TrkA tyrosine phosphorylation by GW441756 impairs the MMP-9 release and, as consequence, the invasive ability of NGF-treated TNBC cells.

Previous findings have reported that TNBC cells might release NGF. As these cells express TrkA, an autocrine loop might sustain their survival (Dollé et al., 2003; Pundavela et al., 2015; Chakravarthy et al., 2016). Our data confirm these results and also indicate that a very low amount of pro-NGF is released by TNBC cells. Although the amount of secreted NGF is lower (almost 4,867 and 6,923 pg/ml for MDA-MB-231 and MDA-MB-453, respectively) than that used (100 ng/ml) to stimulate TNBC cells, it might be argued that a persistent release of NGF, rather than pro-NGF, self-sustains *in vivo* the growth and aggressiveness of TNBC. By a functional assay, we have also verified that CM induces the differentiation of neuronal PC12 cells. Such effect is actually caused by the NGF present in CM since blocking the activity of the secreted NGF by a specific neutralizing antibody reverses the differentiation as well as the NGF-signaling activation of PC12 cells. Thus, once released by TNBC cells, NGF might play a role in the autonomic innervation of the tumor and its aggressiveness. Infiltration of the tumor microenvironment by nerve fibers involves NGF production by BC cells and is associated with BC aggressiveness (Pundavela et al., 2015). Overall, our data might have implications in the brain metastasis of TNBC since neurotrophic factors released by BCs control the interaction between microglial and metastatic BC cells, allowing their growth in the brain (Louie et al., 2013). In this context, the results observed in MDA-MB-231 cells are particularly relevant since they represent a brain-tropic TNBC cell type (Yoneda et al., 2001).

The findings here reported using neutralizing antibodies against NGF or the TrkA inhibitor GW441756 deserve additional comments. Targeting NGF/TrkA signaling by blocking antibodies and/or inhibitors has gained great attention in the last years. Several drugs, including neutralizing antibodies, small inhibitors, and peptides have been synthesized to shut down the NGF circuit in neurological disorders (Longo and Massa, 2013 and references therein). A neutralizing anti-NGF antibody has been used to reduce the pain related to autoimmune diseases and was successfully tested in preclinical models of prostate cancer. A similar approach might be used to alleviate cancer-related pain. Additionally, small molecules targeting NGF signaling are currently used in ongoing clinical trials for the treatment of many solid tumors (Griffin et al., 2018).

The present study, together with our previous findings in prostate cancer (Di Donato et al., 2018, 2019), further points to the relevance of NGF signaling in “gender-related cancers” and paves the way for new therapeutic opportunities in the clinical management of TNBC patients, who often exhibit or develop drug resistance.

## Data Availability Statement

The raw data supporting the conclusions of this article will be made available by the authors, without undue reservation.

## Author Contributions

MDD contributed to the conceptualization, data curation, formal analysis, validation, investigation, and methodology. GG and PG helped with the investigation and methodology. AS reviewed and helped with funding acquisition. AM did the review and editing and helped with funding acquisition. GC contributed to the conceptualization, supervision, funding acquisition, writing original draft, review, and editing. All authors read and approved the final manuscript.

## Conflict of Interest

The authors declare that the research was conducted in the absence of any commercial or financial relationships that could be construed as a potential conflict of interest.
