# Supplementary material for: Targeting the Nerve Growth Factor Signaling Impairs the Proliferative and Migratory Phenotype of Triple-Negative Breast Cancer Cells
Source: Front Cell Dev Biol. 2021 Jun 29;9:676568. doi: 10.3389/fcell.2021.676568 (PMC8275826; doi:10.3389/fcell.2021.676568)

**SUPPLEMENTAL MATERIAL**

**TARGETING THE NERVE GROWTH FACTOR SIGNALING IMPAIRS THE PROLIFERATIVE AND MIGRATORY PHENOTYPE OF TRIPLE-NEGATIVE BREAST CANCER CELLS.**

**Marzia Di Donato°, Giovanni Galasso°, Pia Giovannelli°, Antonio A. Sinisi^, Antimo Migliaccio°# and Gabriella Castoria°#**

*° Dipartimento di Medicina di Precisione- Università della Campania ‘L. Vanvitelli’- Via L. De Crecchio, 7- 80138 Napoli (Italy)*

*^Dipartimento di Scienze Mediche e Chirurgiche Avanzate- Università della Campania ‘L. Vanvitelli’- Via S. Pansini, 5- 80131 Napoli (Italy)*

**
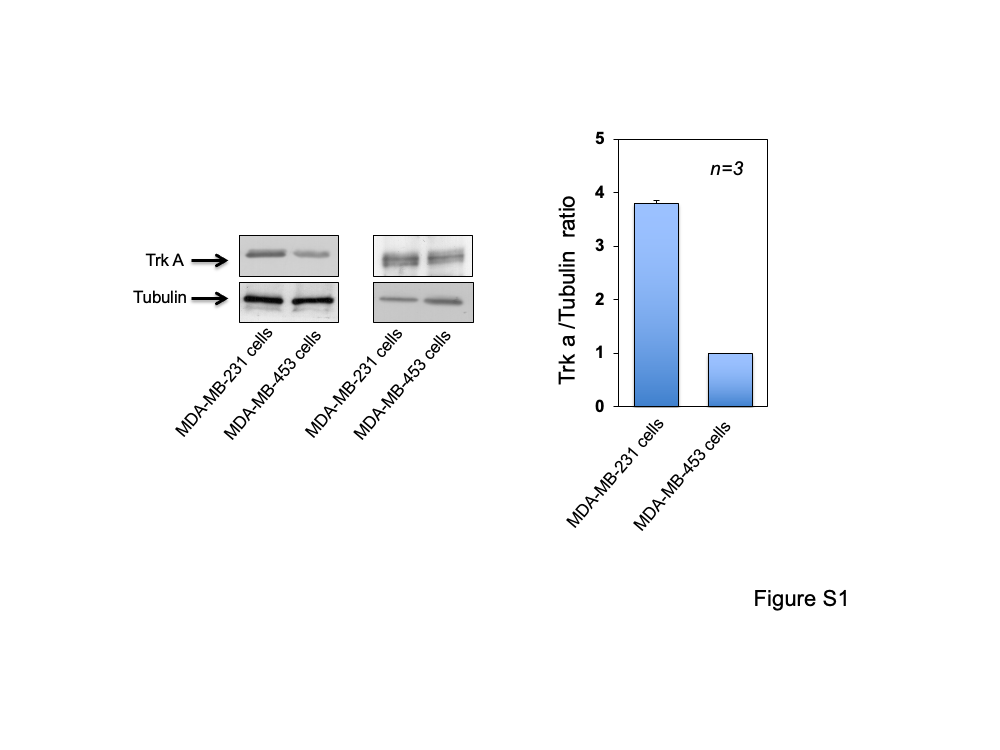
**

**Figure S1.**

Lysate proteins from MDA-MB-231 and MDA-MB453 cells were analyzed by WB, using the anti-TrkA antibody. Two different experiments are presented. Densitometric analysis was done and quantification is shown in the graph. It also includes data from WB presented in the main text (Figure 1A).

**
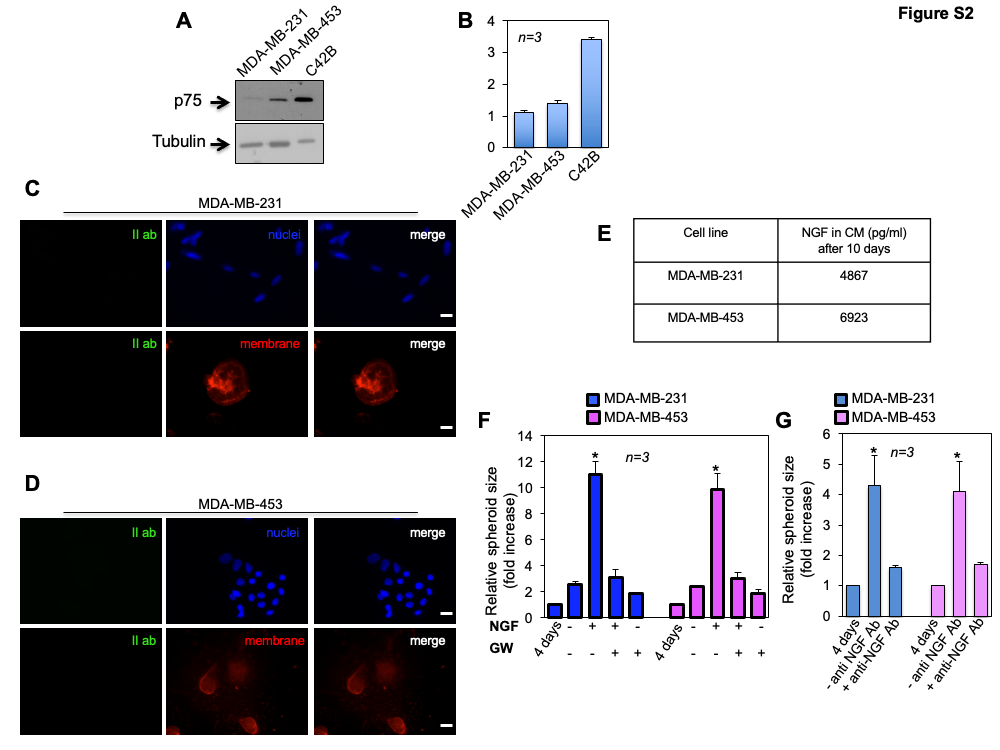
Figure S2.**

MDA-MB-231, MDA-MB453 as well as prostate cancer-derived C4-2B cells were made quiescent. Lysate proteins were prepared and analyzed by WB, using the anti-p75 antibody (**A**). For each sample densitometric analysis was done and quantification is shown in **B**.

Quiescent MDA-MB-231 (**C**) and MDA-MB-453 cells (**D**) on coverslips were stained with the secondary antibody (II ab), nuclei (blue) or plasma membrane (red) and analyzed by IF, as described in Methods. Merged images are shown (right panels). Scale bar, 10μM.

In **E**, MDA-MB-231 and MDA-MB-453 cells were plated and made quiescent. After 10 days, conditioned media (CM) derived by TNBC cells were collected and analyzed for the amount of β NGF (NGF; pg/ml) by ELISA assay.

In **F** and **G**, quantification analysis of TNBC cell-derived spheroids (presented in Figure 3 of the main text) was done and expressed as fold increase over the basal level (at 4^th^ days).


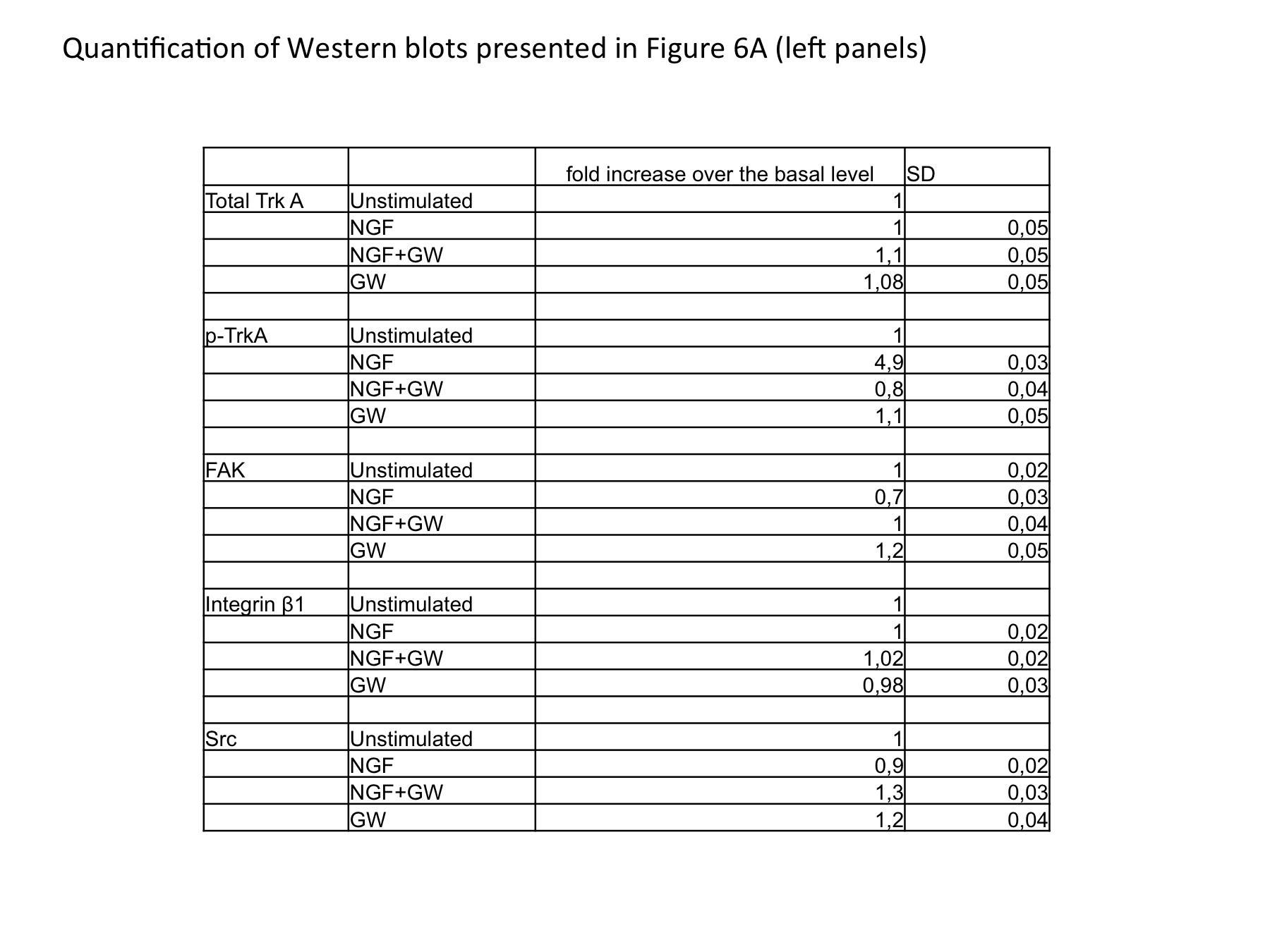


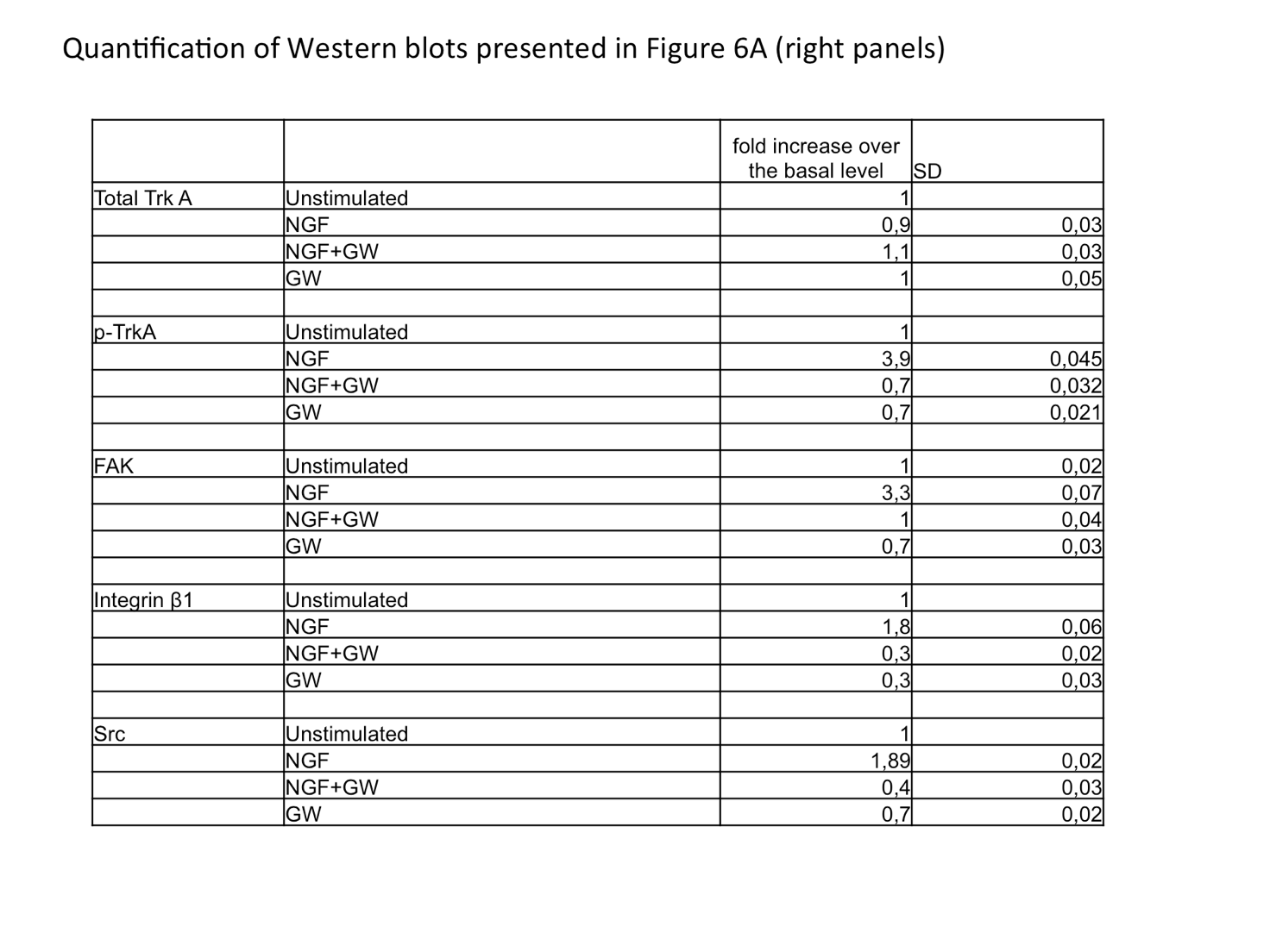


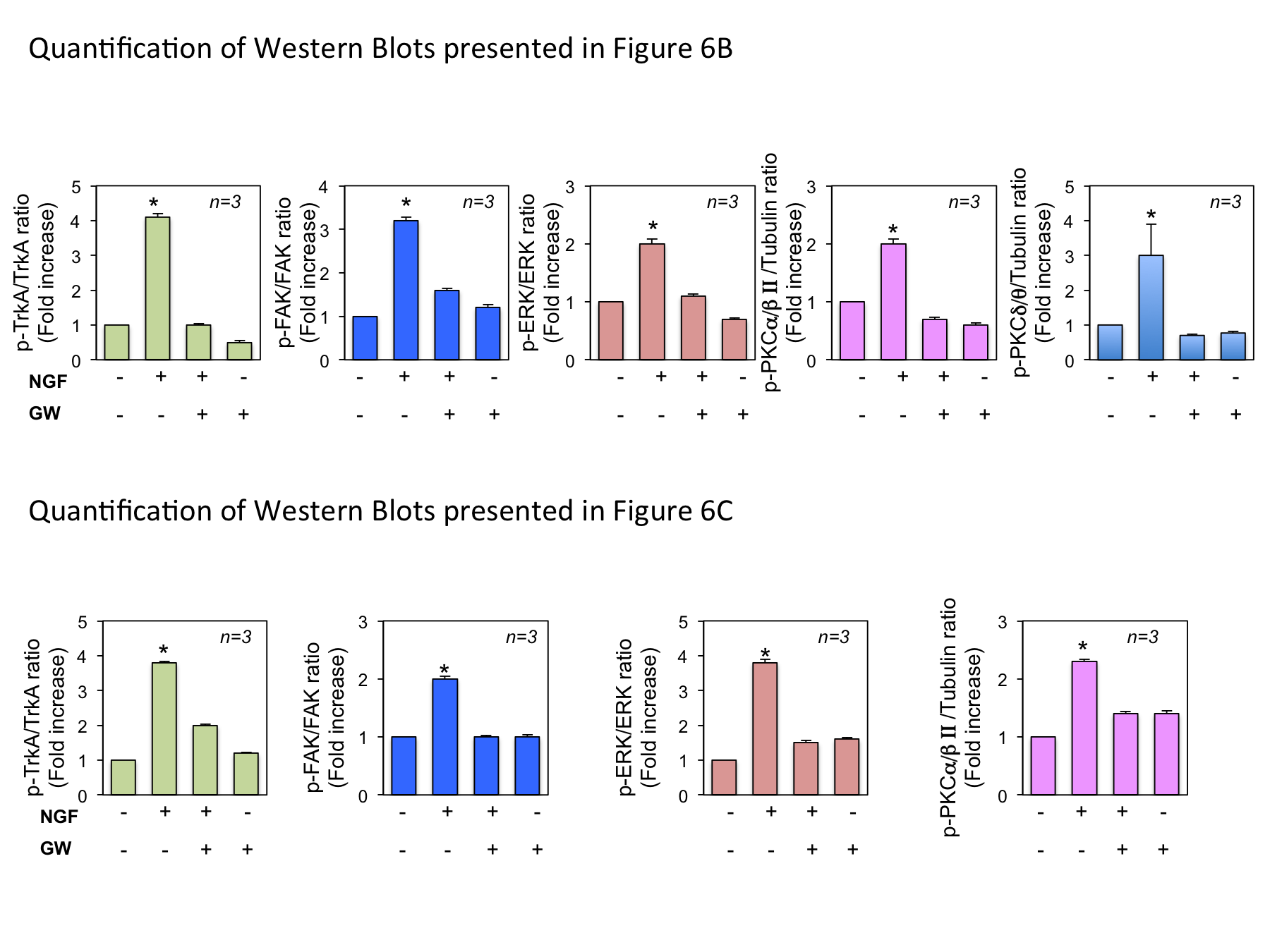

Supplement: Supplementary file 1 [file Table_1.DOCX]
